# Complex endovascular treatment of intact aortic aneurysms: An analysis of health insurance claims data

**DOI:** 10.1007/s00772-018-0387-7

**Published:** 2018-05-07

**Authors:** C.-A. Behrendt, H. C. Rieß, T. Schwaneberg, F. Heidemann, N. Tsilimparis, A.‑A. Larena-Avellaneda, H. Diener, T. Kölbel, E. S. Debus

**Affiliations:** Department of Vascular Medicine, University Heart Center Hamburg, University Medical Center Hamburg-Eppendorf, Hamburg, Germany

**Keywords:** Endovascular procedures, Stroke, Health services research, Survival, Outcome assessment (health care), Endovaskuläre Prozeduren, Schlaganfall, Versorgungsforschung, Überleben, Qualitätsentwicklung

## Abstract

**Background:**

The complex endovascular repair of aortic aneurysms and dissections with fenestrated or branched stent grafts (FB-EVAR) remains challenging for interventional vascular surgery. To date, the evidence regarding treatment patterns and outcome measures consists of single center studies; however, it might be reasonable to validate results with multicenter real-world evidence.

**Methods:**

Health insurance claims data from Germany’s third largest insurance provider, DAK-Gesundheit, were used to determine outcomes following FB-EVAR of non-ruptured thoracic aorta (TA) or thoracoabdominal including pararenal abdominal (TAA) aorta. The study included patients operated between January 2008 and April 2017.

**Results:**

Included were 984 patients (18.1% female) who underwent FB-EVAR. Patients with treatment of the TA were younger (71.7 vs. 73.2 years, *p* < 0.001) and more often female (38.5% vs. 17.0%, *p* < 0.001) as compared to patients with treatment of TAA. In the TA group peripheral arterial disease was less frequent compared to the TAA group (67.3% vs. 80.4%, *p* = 0.036). Mortality was significantly (*p* < 0.001) higher following repair of the TAA compared to the TA at discharge (17.3% vs. 4.6%), at 30 days (26.9% vs. 8.2%) and at 90 days (34.6% vs. 10.1%). Patients with treatment of the TAA suffered more often from stroke as compared to the TA group (7.7% vs. 1.2%, *p* = 0.002).

**Conclusion:**

In this large-scale German analysis of claims data, multicenter real-world evidence was different from single center studies regarding patient risk-factors and outcome measures. Validated multicenter registry studies could help to further investigate this topic in times of increasing procedures.

## Zusammenfassung

**Hintergrund:**

Die komplexe endovaskuläre Versorgung von Aneurysmen und Dissektionen der Aorta mithilfe von fenestrierten oder gebranchten Endoprothesen (FB-EVAR) ist noch immer eine Herausforderung für die endovaskuläre Gefäßchirurgie. Bisher besteht die Evidenzbasis zu diesem Thema weitestgehend aus Single-Center-Studien, die mit einer multizentrischen Versorgungsrealität verglichen werden sollte.

**Methoden:**

Die Daten zu stationären Behandlungen der drittgrößten gesetzlichen Krankenversicherung Deutschlands, DAK-Gesundheit, wurden ausgewertet, um komplexe endovaskuläre Behandlungen der thorakalen (TA) und thorakoabdominellen inklusive der Viszeralgefäße einbeziehenden abdominellen (TAA) Aorta zu analysieren.

**Ergebnisse:**

Zwischen Januar 2008 und April 2017 wurden insgesamt 984 Patienten (18,1 % davon weiblich) endovaskulär mithilfe von fenestrierten oder gebranchten Endoprothesen an der TA oder TAA behandelt. Patienten mit Versorgung der TA waren etwas jünger (71,7 vs. 73,2 Jahre, *p* < 0,001) und häufiger weiblichen Geschlechts (38,5 % vs. 17,0 %, *p* < 0,001) als Patienten mit Versorgung der TAA. Bei TA wurde seltener eine periphere (atherosklerotische) Gefäßerkrankung dokumentiert (67,3 % vs. 80,4 %, *p* = 0,036). Die Krankenhaus- (17,3 % vs. 4,6 %), 30-Tages- (26,9 % vs. 8,2 %) und 90-Tages-Sterblichkeit (34,6 % vs. 10,1 %) war signifikant höher bei Behandlung der TA im Vergleich zur TAA. Die Rate an Schlaganfällen und transienten ischämischen Attacken war höher bei Versorgung der TA (7,7 % vs. 1,2 %, *p* = 0,002) im Vergleich zur Versorgung der TAA.

**Schlussfolgerung:**

In dieser großen Routinedatenanalyse zur Darstellung der multizentrischen Versorgungsrealität zeigten sich relevante Unterschiede sowohl hinsichtlich Patientenalter, Geschlecht und Sterblichkeit zwischen den analysierten Gruppen (TA vs. TAA) als auch im Vergleich mit den derzeit verfügbaren Studienergebnissen. Multizentrische validierte Registerstudien zum Abgleich von Primär- und Sekundärdatenquellen sind zu empfehlen.

## Background

**Fig. 1:**
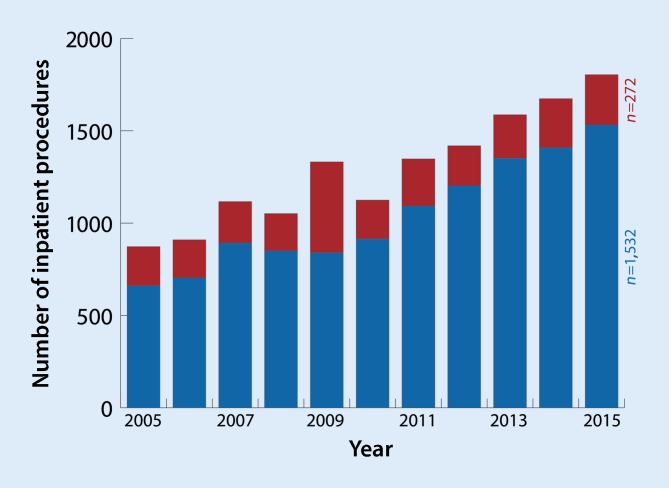
Number of inpatient procedures (case numbers, procedure-specific) Germany-wide in hospital statistics of the German Federal Statistical Office in Wiesbaden (DeStatis) from 2005 to 2015. Thoracoabdominal aortic aneurysm with (*red*) and without (*blue*) evidence of rupture

**Fig. 2:**
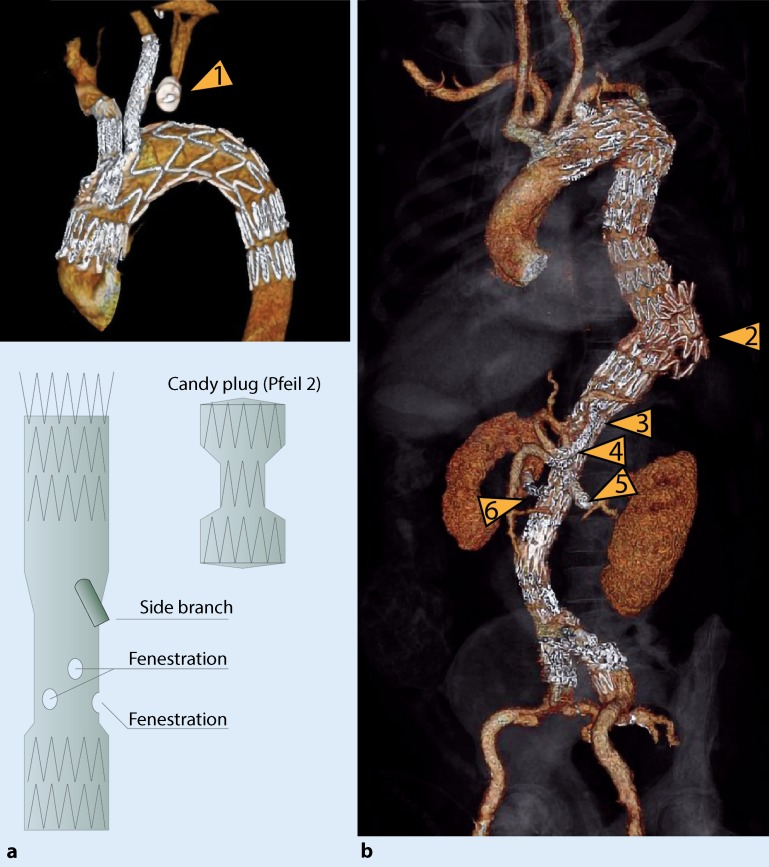
**a** Three-dimensional reconstruction of a branched endograft placed in the aortic arch and occlusion of the left subclavian aorta using a plug (*arrow* *1*). **b** Three-dimensional reconstruction of a long four-branched thoracoabdominal endograft with overstenting and occlusion of the left subclavian artery (*arrow* *2* candy plug placed in the false lumen, *arrow* *3* celiac artery, *arrow* *4* superior mesenteric artery, *arrow* *5* left renal artery, *arrow* *6* right renal artery)

Aortic aneurysms and dissections that may involve supra-aortic or visceral branches and which require treatment are of central importance in interdisciplinary vascular medicine. As such, their epidemiology [29] as well as their treatment have changed fundamentally over the last few decades [27]. The statistics on procedure-specific diagnosis related groups (DRG) compiled by the German Federal Statistical Office (*Statistisches Bundesamt*, DeStatis) in Wiesbaden have for years been showing a rising number of annual procedures coded for thoracoabdominal pathologies (Fig. 1; [12]). Besides the strictly infrarenal or thoracic aortic aneurysms that do not involve the visceral segment or supra-aortic branches, these complex pathologies represent a particular challenge in interventional vascular surgery [2]. This entity, as well as its successful open management, was first described as early as 1955 by the vascular surgeon Stephen N. Etheredge (Oakland, California) [16]. Thoracoabdominal aneurysms can be classified into types I–V according to the Crawford classification (modified according to Safi) [11, 26]. Today, a variety of minimally invasive procedures are available for endovascular aortic repair (EVAR), (Fig. 2), whereas 15 years ago complex aneurysm repair was mostly still performed in an open procedure (open aortic repair, OAR). Technical advances in the endografts available also result in increased demands on the surgeon’s interventional experience and the infrastructure of the treating center. Against the backdrop of an ever-aging population with increasing life expectancy, this progress is the subject of controversy. This original article provides an overview of complex endovascular repair of intact aortic aneurysms and aortic dissections in German hospitals using claims data from the third largest German statutory health insurance, *DAK-Gesundheit* (DAK-G).

## Methods

### Study population and statistics

The database of the DAK-G, Germany’s third largest statutory health insurance (SHI), contains all outpatient and inpatient procedures performed on 6.5 million insured persons (accounting for 8% of all inhabitants in Germany). The DAK‑G database has previously been used for studies on abdominal aortic aneurysms (AAA) [7, 30], Lyme disease [23], skin cancer [1], and severe psychiatric disorders [17]. The DAK-G data can be used to create a population reference to the SHI population, showing comparable gender and age distributions (40.4% female, 29.1% ≥ 65 years).

All claims for inpatient hospital treatment according to § 301 and § 115 of the German Social Code (*Sozialgesetzbuch*, SGB) V submitted between January 2008 and April 2017 with the World Health Organization (WHO) International Classification of Diseases 10 (ICD-10) diagnosis of thoracic (TA, I71.1, I71.2), thoracoabdominal (TAA, I71.5, I71.6), or abdominal (AAA, I71.3, I71.4) aortic aneurysm, or with the WHO ICD-10 diagnosis of thoracic (dTA, I71.01, I71.05), thoracoabdominal (dTAA, I71.03, I71.07), or abdominal (dAA, I71.02, I71.06) aortic dissection or to which a German operation and procedure key (*Operationen- und Prozedurenschlüssel*, OPS) for a complex endovascular aortic repair was coded (Table 1), were included in the selection. Patients diagnosed with rupture were subsequently excluded from further analysis, thereby ensuring that only intact aneurysms or dissections were considered.

The patient selection only considers intact aneurysms or dissections

The patient selection only considers intact aneurysms or dissections based on the localization of endovascular repair, the study population was divided into thoracic procedures (TA, complex aortic arch repair) and thoracoabdominal procedures (TAA). Abdominal aortic procedures involving the visceral vessel segment were assigned to the TAA group. The German OPS code is based on the international classification of procedures in medicine (ICPM). Administrative and demographic data (age, gender), primary and secondary procedures, case-based diagnoses as well as reasons for discharge were collected for all cases identified. The first procedure submitted was deemed an index procedure. The Elixhauser comorbidity index [15, 25], which enables the uniform classification of WHO ICD-10 codes into 30 categories, was used to measure comorbidity. The linear comorbidity score according to van Walraven et al. [31] was then used to create a metric covariate from the coded comorbidities (−19 to +89).

**Table 1:** OPS codes from the reported years 2008–2016 for the selection of complex procedures

	OPS codes (complex procedures)
Thoracic procedures (aortic arch)	*5–38a.7b, 5–38a.7c, 5–38a.7d, 5–38a.7e, 5–38a.7f, 5–38a.72, 5–38a.73, 5–38a.74, 5–38a.75, 5–38a.76, 5–38a.77, 5–38a.78, 5–38a.79, 5–38a.7a*
Thoracoabdominal procedures	*5–38a.8c, 5–38a.8d, 5–38a.8e, 5–38a.8* *f, 5–38a.8g, 5–38a.8h, 5–38a.81, 5–38a.82, 5–38a.83, 5–38a.84, 5–38a.85, 5–38a.86, 5–38a.87, 5–38a.88, 5–38a.89, 5–38a.8a, 5–38a.8b*
Abdominal procedures	*5–38a.c1, 5–38a.c2, 5–38a.c3, 5–38a.c4, 5–38a.c5, 5–38a.13, 5–38a.16, 5–38a.17, 5–38a.18, 5–38a.19, 5–38a.1a, 5–38a.1b, 5–38a.1c, 5–38a.1d, 5–38a.1f, 5–38a.1g, 5–38a.1h, 5–38a.1j, 5–38a.1k, 5–38a.1m, 5–38a.1n, 5–38a.1p, 5–38a.1q, 5–38a.1r, 5–38a.1s, 5–38a.1t, 5–38a.1u, 5–38a.1v, (additional code: 5–38a.w)*

### Ethical aspects

Since the project is a retrospective analysis of anonymized statutory health insurance parameters collected in the context of routine procedures, it does not represent research on humans and does not fall under research projects requiring consultation. Therefore, in accordance with applicable case law, no ethics approval is required and patient consent was not obtained. The study group is not able to identify individual subjects on the basis of the available data.

## Results

According to the DAK database, 984 patients underwent complex endovascular repair for intact aortic aneurysms between January 2008 and April 2017. In total, 52 cases (5.3%) of isolated TA involving supra-aortic vessels (aortic arch) were treated, while 932 cases (94.7%) of TAA or abdominal aorta involving visceral vessels were treated. Table 2 shows patient characteristics and risk factors. The average patient age was 71.7 years at the time of TA repair and 73.2 years at TAA repair (*p* < 0.001). The percentage of male patients was lower in the TA group (61.5% vs. 83.0% for TAA, *p* < 0.001). With the exception of a higher rate of peripheral vascular disease (80.4% vs. 67.3%, *p* = 0.036) in the TAA group, there were no significant differences in terms of comorbidities. At 6.46 and 6.86 points (*p* = 0.689), respectively, the van Walraven comorbidity index was comparable in the two groups.

**Table 2:** Patient characteristics of patients undergoing complex thoracic (TA, *n* = 52) and thoracoabdominal (TAA,* n* = 932) repair

	TA (*N* = 52)	TAA (*N* = 932)	*p*-value
*Age, *years, MV (SD)	*71.67 (8.26)*	*73.16 (7.88)*	*<0.001*
Female* gender, n *(%)	*20 (38.5)*	*158 (17.0)*	*<0.001*
vW* comorbidity index, *MV (SD)	6.46 (6.84)	6.86 (6.91)	0.689
*Heart failure, n *(%)	1 (1.9)	106 (11.4)	0.057
*Cardiac arrhythmia, n *(%)	10 (19.2)	162 (17.4)	0.878
*Cardiac valve disease, n *(%)	3 (5.8)	58 (6.2)	1.0
Peripheral* vascular disease, n *(%)	*35 (67.3)*	*749 (80.4)*	*0.036*
*Hypertension, n* (%)	29 (55.8)	580 (62.2)	0.431
*COPD, n *(%)	8 (15.4)	132 (14.2)	0.967
*Diabetes, *uncomplicated*, n *(%)	3 (5.8)	100 (10.7)	0.366
*Diabetes, *complicated*, n *(%)	1 (1.9)	36 (3.9)	0.733
*Kidney failure, n *(%)	9 (17.3)	247 (26.5)	0.191
*Liver disease, n *(%)	2 (3.8)	15 (1.6)	0.511
*Gastric ulcer, n *(%)	0 (0.0)	1 (0.1)	1.0
*Overweight, n *(%)	5 (9.6)	94 (10.1)	1.0
*Depressive disorders, n *(%)	0 (0.0)	16 (1.7)	0.697

*SD* standard deviation, *MV* mean value, *COPD* chronic obstructive pulmonary disease, *vW* van Walraven, *TA* thoracic aortic aneurysm, *TAA* thoracoabdominal aortic aneurysm

Table 3 shows hospital mortality and relevant treatment outcomes. The median hospital stay was 14 days for TA repair and 10 days for TAA repair (*p* = 0.057). The hospital, 30-day, and 90-day mortality rates were 17.3%, 26.9%, and 34.6%, respectively, for TA repair and 4.6%, 8.2%, and 10.1%, respectively, for TAA repair (*p* < 0.001). The rate of stroke and transient ischemic attack was significantly higher following TA repair (7.7% vs. 1.2%, *p* = 0.002).

The annual number of inpatient treatment cases is continuously rising

In total, 40 patients (7.7% in the TA group and 3.9% in the TAA group, *p* = 0.319) were transferred to another hospital following treatment. Hospital readmission was necessary in the further course in 3.8% and 2.7% of patients, respectively, while repeat surgery was performed in 86.5% and 75.5% of patients, respectively.

**Table 3:** Treatment outcomes for patients with complex thoracic (TA, *n* = 47) and thoracoabdominal (TAA, *n* = 902) repair

	TA (*N* = 52)	TAA (*N* = 932)	*p*-Value
*Hospital mortality, n *(%)	*9 (17.3)*	*43 (4.6)*	*<0.001*
*30-day mortality, n *(%)	*14 (26.9)*	*76 (8.2)*	*<0.001*
*90-day mortality, n *(%)	*18 (34.6)*	*94 (10.1)*	*<0.001*
*Hospital stay, *days, MV (SD)	17.9 (13.7)	14.3 (13.2)	0.057
*Hospital stay, *days, median	14	10	
Transfer to *another hospital, n *(%)	4 (7.7)	36 (3.9)	0.317
Discharge to* rehabilitation, n *(%)	2 (3.8)	25 (2.7)	0.949
*Inpatient readmission, n *(%)	2 (3.8)	23 (2.5)	0.871
*Re-operation *in the further course*, n* (%)	45 (86.5)	704 (75.5)	0.1
Acute* respiratory insufficiency, n *(%)	10 (19.2)	96 (10.3)	0.073
*Pneumonia, n *(%)	4 (7.7)	33 (3.5)	0.247
Acute* kidney failure, n *(%)	5 (9.6)	75 (8.0)	0.887
Acute* renal infarction, n *(%)	0 (0)	15 (1.6)	0.734
Acute* myocardial infarction, n *(%)	1 (1.9)	22 (2.4)	1.0
*Stroke or TIA, n *(%)	*4 (7.7)*	*11 (1.2)*	*0.002*
Acute* intestinal ischemia, n *(%)	3 (5.8)	16 (1.7)	0.121
*Ischemia of the extremities, n *(%)	3 (5.8)	37 (4.0)	0.781
*Amputation, n *(%)	0 (0)	3 (0.3)	1.0
*Paraplegia, n *(%)	3 (5.8)	26 (2.8)	0.415
*Hemorrhage, n *(%)	18 (34.6)	255 (27.4)	0.328
*Gastric ulcer, n *(%)	0 (0)	12 (1.3)	0.862
*Sepsis or SIRS, n *(%)	2 (3.8)	16 (1.7)	0.56

*SD* standard deviation, *MV* mean value, *SIRS* systemic inflammatory response syndrome, *TIA* transient ischemic attack, *TA* thoracic aortic aneurysm, *TAA* thoracoabdominal aortic aneurysm

**Fig. 3:**
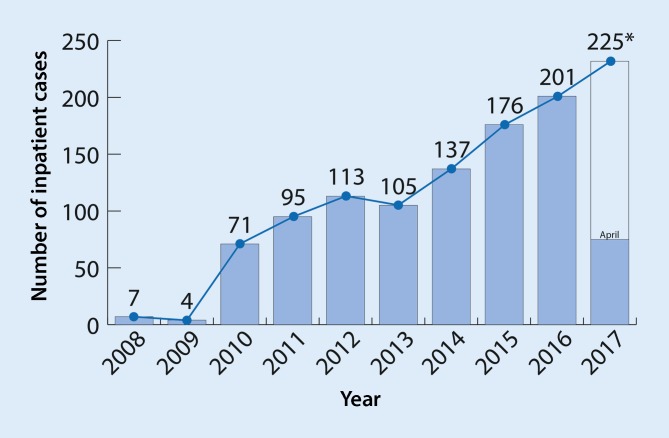
Inpatient cases involving complex endovascular repair of thoracic and thoracoabdominal aortic aneurysms (TA, TAA) claimed for between January 2008 and April 2017 (*case number prognosis)

A continuous rise was seen throughout the study period in the annual number of cases of inpatient treatment (from 7 in 2008 to 201 in 2016; proportionately 75 to April 2017). This corresponds to an absolute increase of more than 2800% between 2008 and 2016 and 283% between 2010 and 2016 (Fig. 3).

## Discussion

This large-scale German analysis of claims data on complex endovascular repair of aortic diseases is the first study to analyze a database that provides an insight into the actual situation in terms of multicenter care. It demonstrates that there are significant differences between TA and TAA care in terms of age and gender distribution, short-term and medium-term mortality, and complications.

The endovascular treatment of aortic diseases that may involve visceral or supra-aortic vessels remains a challenge in modern vascular surgery. The evidence available on risk factors and treatment outcomes is largely based on single-center case series (Table 4). Due to possible selection and publication bias, as well as the unknown external and internal validity of these data, a comparison of the results with large registry or claims data is useful. On the whole, the patient characteristics and endpoints of the published case series of 1569 patients from 8 single center studies vary considerably. While the technical success in the case series was consistently high at 92–100%, the 30-day mortality rate among patients treated between 2001 and 2016 was between 0% and 6.2%. There were also marked differences between the respective cohorts included in terms of patient age (70.5–75 years) and the proportion of male patients (47–93.8%) (Table 4). These differences make it likely that relevant confounders were present. Mastracci et al. (610 type IV thoracoabdominal aortic aneurysms, TAAA) [22] and Eagleton et al. (354 type II and type III TAAA) [13] published the results of the largest study in terms of numbers with the longest post-interventional follow-up. Technical success was 97% and 94.1%, respectively, with a 30-day mortality rate of 4.8% for type II to type III TAAA. Aneurysm-related mortality was as low as 2% at 8 years following type IV TAAA repair. A total of 18.6% of patients with type II to type III TAAA had pre-existing kidney failure. Acute kidney failure was detected following intervention in 5.1% of patients and permanent spinal ischemia in 4% [13]. The results of 100 cases of consecutive endovascular repair of complex abdominal aortic aneurysms (AAA, including iliac findings) and TAAA were reported in the most recent prospective single center analysis by Schanzer et al. [28]. The average hospital stay in this case series was only 3.6 days. At 30 days, 3% of patients had died and intestinal ischemia was seen in 1% of cases. Paralysis, heart attack, and stroke were not observed [28]. Another single center analysis conducted by Budtz-Lilly et al. demonstrated a 30-day mortality rate of 2.8% and a 90-day mortality rate of 9.9% based on the retrospective data of 71 consecutively treated patients. In all, 15.0% (juxtarenal AAA) and 22.6% (TAAA) of patients had chronic kidney failure prior to intervention. Permanent post-procedural spinal damage was observed in only 2.8% of patients [9].

**Table 4:** Overview of the case series in the literature on complex endovascular repair of aortic pathologies

Author	Period	Number of cases	Centers	Pathologies, treatment	Outcomes
Schanzer et al. 2017 [28]	2010–2016	*n* = 100	Single center	Complex repair of iliac bifurcation, juxtarenal, pararenal, and TAAA (types I–IV)	89% technical success, 3% mortality at 30 days
		(75 years, 68% males)			
Piffaretti et al. 2017 [24]	2006–2016	*n* = 17 (of 283 evaluated)	Single center	Elective TEVAR including celiac artery	100% technical success, 0% hospital mortality
		(74 years, 47% males)			
Budtz-Lilly et al. 2017 [9]	2010–2015	*n* = 71	Single center	Juxtarenal and pararenal AAA, type II–IV TAAA, elective and emergency, f‑EVAR, b‑EVAR	95–96% technical success, AAA: 2.5% mortality at 30 days TAAA: 3.7% mortality at 30 days
		AAA: (73 years, 85% males), TAAA: (70 years, 48.4% males)			
Eagleton et al. 2016 [13]^a^	2004–2013	*n* = 354	–	Type II and III TAAA, elective f‑EVAR, b‑EVAR	94.1% technical success, 4.8% mortality at 30 days
		(73.5 years, 76.3% males)			
Martin-Gonzales et al. 2015 [21]	2004–2012	*n* = 225	Single center	Type I–V TAAA, elective f‑EVAR, b‑EVAR	95.5% technical success, 6.2% mortality at 30 days
		(70.5 years, 93.8% males)			
Mastracci et al. 2015 [22]^a^	2001–2013	*n* = 610	–	Type IV TAAA (*n* = 349), juxtarenal (*n* = 258), unclassified (*n* = 3), f‑EVAR, b‑EVAR	95–96% technical success, 2% aneurysm-related fatalities at 8 years
		(75 years, 82.1% males)			
Kristmundsson et al. 2014 [19]	2002–2007	*n* = 54	Single center	f-EVAR	3.7% Surgical mortality
		(72 years, 85% males)			
Grimme et al. 2014 [18]	2001–2011	*n* = 138	Single center	Branched, fenestrated	92% Technical success, 1.4% mortality at 30 days
		(73 years, 89.1% males)			

^a^study population possibly also partially described in other publications in this table

Our current analysis of claims data cannot readily confirm the results of the abovenamed single center analyses and case series. A possible selection bias is already evident in terms of the age and gender distribution. Patients in the single center trials were somewhat older and, with one exception, more frequently male compared with this study population. Closer scrutiny of the disparately defined comorbidities in the various study populations revealed other relevant differences. Whereas there is acceptable concordance in the rates of diabetes, cardiac arrhythmia, and chronic kidney disease between the different studies, significant differences are seen particularly in peripheral vascular diseases (e. g., peripheral arterial occlusive disease, coronary heart disease, and carotid stenosis). For example, chronic obstructive pulmonary diseases (COPD) are significantly more rarely coded in the DAK database compared with the primary data sources (Table 5). The validity of data from non-quality assured registries and claims data sources has recently been the subject of regular controversy [8, 32]. Projects designed to validate the data are also limited due to differing definitions of data collection parameters. In this context, the use of the Elixhauser comorbidity classification (into a total of 30 different groups) in this study improves comparability between different administrative records and WHO coding systems [14, 15, 25].

**Table 5:** A comparison of various (differently defined) risk factors in this study with the single center studies

Author	CHF (%)	CA (%)	PVD (%)	AHTN (%)	COPD (%)	DM (%)	CKD (%)
This study	10.9	17.5	79.7	61.9	14.2	14.2	26.0
Schanzer et al. 2017 [28]	–	–	55 (CHD)	85	29	14	26
Piffaretti et al. 2017 [24]	–	18 (AF)	12 (CHD)	100	53	12	12
Budtz-Lilly et al. 2017 [9]	12.7	21.1 (AF)	39.4	83.1	31.0	5.6	18.3
Eagleton et al. 2016 [13]^a^	–	24.9	43.8	–	30.8	14.7	18.6
Martin-Gonzales et al. 2015 [21]	5.8	14.7	50.7 (CAD)	79.1	42.2	20.9	23.6
			35.6 (PAD)				
Mastracci et al. 2015 [22]^a^	–	27.9	–	–	31.1	19.5	–
Kristmundsson et al. 2014 [19]	–	–	–	–	–	–	50
Grimme et al. 2014 [18]	–	–	69.5 (CAD)	87.6	48.9	15.2	35.5

*CHF* chronic heart failure, *CA* cardiac arrhythmia, *PVD* peripheral vascular disease, *AHTN* arterial hypertension, *COPD* chronic obstructive pulmonary disease, *DM* diabetes mellitus, *CKD* chronic kidney disease

^a^study population possibly also partially described in other publications in this table

A further limitation in terms of valid comparability arises from the studies’ different inclusion periods. The question of whether improved generations of products, the introduction of new procedures and techniques, and the individual interventionalist’s learning curve as possible influencing factors has long been discussed [10, 20]. If one looks at the marked rise in the annual number of cases (Fig. 3), it becomes apparent that the reality of nationwide medical care in 2010, with around one third of today’s annual case numbers, cannot be easily compared across the board with the situation in 2017. To this one can add the rising number of previously treated patients in whom a higher rate of post-interventional complications (e. g., spinal ischemia) can be expected.

The treatment reality from 2010 is not comparable with the situation in 2017

Against this background, the question arises as to which criteria can be used to obtain informed consent from suitable patients and which information can be passed on to patients in an evidence-based manner. Since single center analyses that lack independent data monitoring and validation tend to have system-related selection and publication biases, independent sources of data are required in order to make comparisons with the reality of nationwide medical care. Although claims data can possibly close this gap, they in turn are subject to relevant limitations.

### Limitations

Since DAK-G claims data are primarily collected for administrative and reimbursement purposes, conscientious data validation and quality assurance is required for their secondary use [6]. Internal validity varies and is generally greater for reimbursement-relevant codes than for codes that are not relevant to reimbursement. In the meantime, study projects such as the VISION initiative in the USA or the IDOMENEO study in Germany are addressing in greater detail the validity of claims data in vascular outcome assessment and treatment research [5]. Due to their better external validity compared with registry data, claims data are also suitable for analyzing rare events or treatments, such as in complex aortic pathologies. In contrast to registry surveys, where the treating physician often decides which data are submitted, the collection of claims data is not limited to isolated diseases, individual specialist disciplines or the duration of hospital stay. Particularly in the case of group comparisons, one can also assume that so-called overcoding for reimbursement reasons occurs in both groups to the same extent; as such, the results obtained could still be valid. Naturally, the analysis of claims data cannot replace randomized controlled trials (RCT); however, collecting supplementary data and comparing RCTs with the reality of medical care can provide important insights.

## Conclusion

This large-scale analysis of claims data to demonstrate the actual situation in multicenter care revealed relevant differences not only in terms of patient age, gender, and mortality between the groups analyzed (TA vs. TAA), but also in comparison with the study results currently available. The significantly higher stroke rate in complex endovascular TA repair is also worthy of note. Multicenter, validated registry studies to compare primary and secondary data sources are recommended.
